# Accountability mechanisms for implementing a health financing option: the case of the basic health care provision fund (BHCPF) in Nigeria

**DOI:** 10.1186/s12939-018-0807-z

**Published:** 2018-07-11

**Authors:** Benjamin Uzochukwu, Emmanuel Onwujekwe, Chinyere Mbachu, Chinyere Okeke, Sassy Molyneux, Lucy Gilson

**Affiliations:** 1 Department of Community Medicine, College of Medicine, University of Nigeria, Enugu-Campus, Enugu, Nigeria; 2 Institute of Public Health, University of Nigeria, Enugu-Campus, Enugu, Nigeria; 3 Department of Health Administration and Management, College of Medicine, University of Nigeria, Enugu-Campus, Enugu, Nigeria; 4 Health Policy Research Group, College of Medicine, University of Nigeria, Enugu-Campus, Enugu, Nigeria; 5 KEMRI-Wellcome Trust Research Programme, Kilifi, Kenya; 6 Health Policy and Systems Division, School of Public Health and Family Medicine, University of Cape Town, Cape Town, South Africa

**Keywords:** Accountability mechanisms, Basic health care provision fund, Equity, Nigeria

## Abstract

**Background:**

The Nigerian National Health Act proposes a radical shift in health financing in Nigeria through the establishment of a fund – Basic Healthcare Provision Fund, (BHCPF). This Fund is intended to improve the functioning of primary health care in Nigeria. Key stakeholders at national, sub-national and local levels have raised concerns over the management of the BHCPF with respect to the roles of various stakeholders in ensuring accountability for its use, and the readiness of the implementers to manage this fund and achieve its objectives. This study explores the governance and accountability readiness of the different layers of implementation of the Fund; and it contributes to the generation of policy implementation guidelines around governance and accountability for the Fund.

**Methods:**

National, state and LGA level respondents were interviewed using a semi structured tool. Respondents were purposively selected to reflect the different layers of implementation of primary health care and the levels of accountability. Different accountability layers and key stakeholders expected to implement the BHCPF are the Federal government (Federal Ministry of Health, NPHCDA, NHIS, Federal Ministry of Finance); the State government (State Ministry of Health, SPHCB, State Ministry of Finance, Ministry of Local Government); the Local government (Local Government Health Authorities); Health facilities (Health workers, Health facility committees (HFC) and External actors (Development partners and donors, CSOs, Community members).

**Results:**

In general, the strategies for accountability encompass planning mechanisms, strong and transparent monitoring and supervision systems, and systematic reporting at different levels of the healthcare system. Non-state actors, particularly communities, must be empowered and engaged as instruments for ensuring external accountability at lower levels of implementation. New accountability strategies such as result-based or performance-based financing could be very valuable.

**Conclusion:**

The key challenges to accountability identified should be addressed and these included trust, transparency and corruption in the health system, political interference at higher levels of government, poor data management, lack of political commitment from the State in relation to release of funds for health activities, poor motivation, mentorship, monitoring and supervision, weak financial management and accountability systems and weak capacity to implement suggested accountability mechanisms due to political interference with accountability structures.

## Background

Good health system governance is characterized by responsiveness and accountability. The extent to which actors interact in governance, as well as the institutional, bureaucratic and social factors that influence these interactions, all work together to ensure health system accountability. Accountability features prominently in all governance definitions, either as a key function or outcome and improved accountability is often called for as an element in improving health system performance. Three general categories of accountability have been noted: financial, performance, and political/democratic accountability [1]. Financial accountability concerns tracking and reporting on allocation, disbursement, and utilization of financial resources, using the tools of auditing, budgeting, and accounting and focuses on the control of the misuse and abuse of public resources and/or authority. Performance accountability deals with supporting improved service delivery and management through feedback and learning and focuses primarily on services, outputs, and results while political/democratic accountability has to do with the institutions, procedures, and mechanisms that ensure that government delivers on electoral promises.

All health systems contain accountability relationships of different types, which function with varying degrees of success. In addition to the three general categories of accountability, an important broad distinction is between ‘external’ accountability mechanisms which may be used by non-state actors to hold public sector power-holders to account, and ‘internal’ accountability mechanisms that are comprised of the institutional oversights, checks and balances internal to the public sector [1, 2]. Accountability mechanisms therefore are governance tools which seek to regulate answerability between the health system and / or citizens and between different levels of the health system [3].

The concern with accountability and health systems originates from the dissatisfaction with health system performance, availability and equitable distribution of basic services, abuses of power, financial mismanagement and corruption, and lack of responsiveness [4]. In addition, proper accounting for the use of primary health care (PHC) funds is a high priority for both governments and donors because of the importance of PHC in delivering health care to the majority of the populace. Therefore, strengthened accountability has been recommended as a remedy for strengthening health system weaknesses around the world [4].

In October 2014, following a decade of planning, the Nigerian President signed into law the National Health Act (NHAct). The Act, provides a legal framework for the provision of health care services to all Nigerians and for the organisation and management of the health system. This could not have come at a better time as Nigeria currently has some of the worst health outcomes in the world, due in part to the poor state of primary health care services. For example the 2013 National Demographic Health Survey (NDHS) shows that the common preventable diseases like malaria, diarrhoea and malnutrition are major causes of morbidity and mortality in children; maternal mortality is 576/100,000 and U5MR is 69/1000 live births with life expectancy of 52.62 years. Antenatal care attendance and delivery by skilled health providers were 61 and 38% respectively; and only about a quarter of children were fully vaccinated.

A key component of the NHAct is the establishment of the Basic Health Care Provision Fund (BHCPF) which will be predominantly financed through an annual grant from the Federal Government of not less than 1 % of the Consolidated Revenue Fund (CRF) which is the total Federal Revenue before it is shared to all tiers of government. Based on the draft Medium Term Expenditure Framework and Fiscal Strategy Paper (MTEF&FSP) 2017–2019, the BHCPF (1% of CRF) translates to an average of N35bn per annum or 114.7 million US dollars per annum as at 2016. Half of the Fund will be used to provide a basic package of services in PHC facilities through the National Health Insurance Scheme (NHIS); 45% will be disbursed by the National Primary Health Care Development Agency (NPHCDA) for essential drugs, maintaining PHC facilities, equipment and transportation, and strengthening human resource capacity; and the final 5% will be used by the Federal Ministry of Health (FMOH) to respond to health emergencies and epidemics. Additional sources of funding for the BHCPF could include grants by international donors and funds generated from innovative sources such as taxes on cigarettes and alcohol. Further, to be eligible for Fund donations, States and Local government areas are expected to contribute 25% counterpart funding respectively towards PHC projects. It is expected that provision of this fund will ensure that quality primary health care provisions are affordable and accessible to all and thus equitable.

### Governance & Health service organization in Nigeria

The Country operates a federal system of government comprising 36 States and the Federal Capital Territory. The health system in Nigeria is based on the three tier structure of government (Federal, State and Local Government Authority (LGA) each with substantial autonomy. Every state and local government has a State Ministry of Health (SMOH) and Local Government Health Department respectively. However, the roles and responsibilities of the different levels of the health system with respect to PHC are unclear. The overlaps in roles often result in duplication of efforts and wastage on one spectrum, or total neglect of roles [5]. The federal government through the Federal Ministry of Health (FMOH) is primarily responsible for overall stewardship and leadership for health. The SMOH provides health care services through secondary level health facilities as well as technical assistance to the Local Government Area Health Departments. LGAs own and fund PHC facilities and have overall responsibility for this level of care with the health posts and clinics, health centers and comprehensive health centers providing basic primary care services. A dynamic private sector offers an opportunity to fill part of the gap left by a weak PHC system. However, health equity is not very high on the policy agenda, thus creating a problem of affordability and accessibility among the poor and the less advantaged groups [5].

Financing lies at the core of Nigeria’s PHC delivery challenges. The PHC budget at the Federal level has been decreasing over the past four years. It decreased from 8.4% of total spending in the health sector in 2012 to 4.7% in 2015 [6]. At the LGA levels, the financial allocations do not extend beyond the payment of salaries and budgets are not earmarked, leading to delays in the release (or at times non-release) of PHC funds. Accountability and transparency are some of the weakest areas of the public finance system in Nigeria, particularly at this level.

**Fig. 1:**
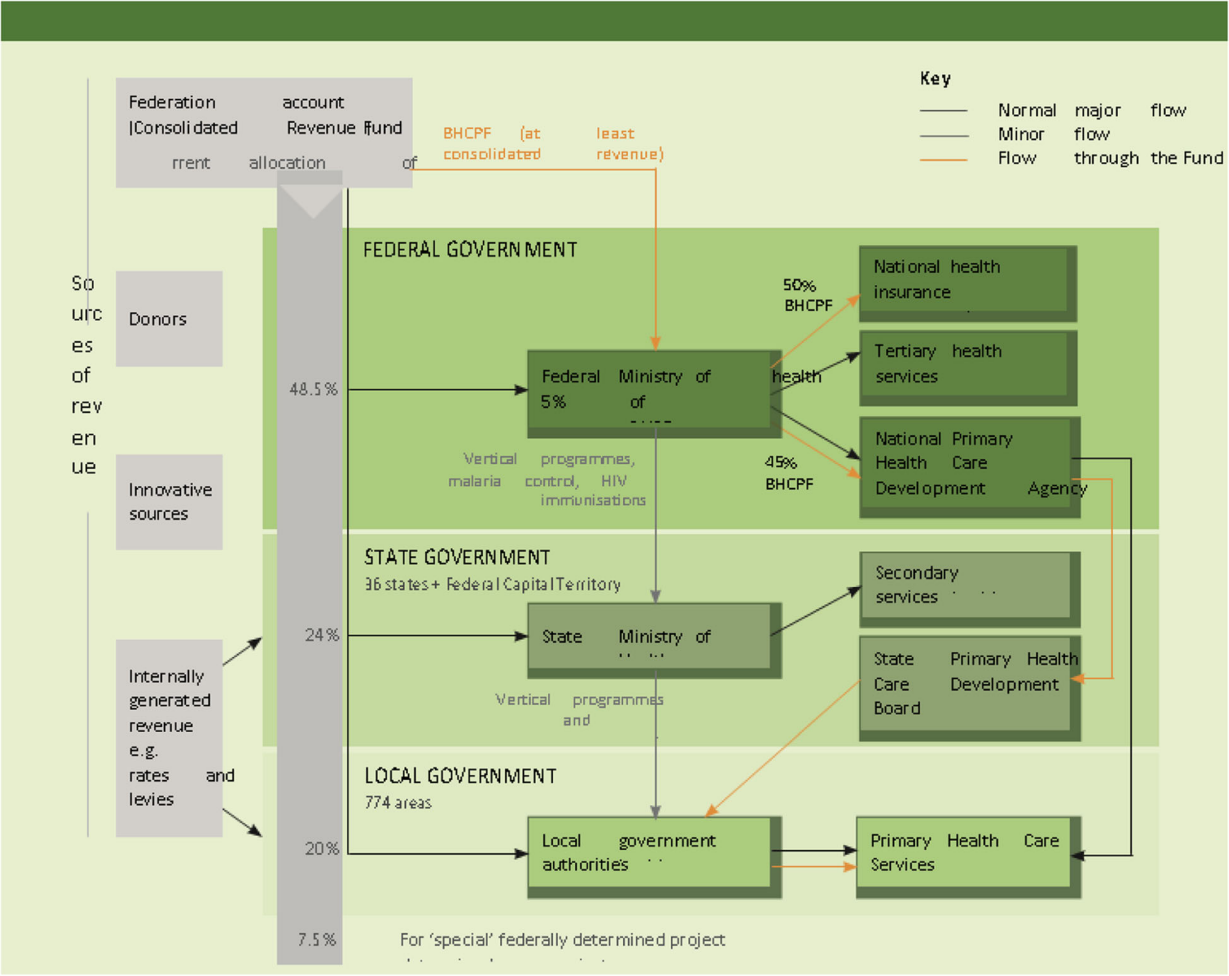
Flows of funds from health services

Figure 1 shows the current flow of funds for health services in Nigeria and the proposed flow of revenue of the BHCPF. The solid and dashed arrows show the normal and minor flows respectively while the yellow arrow shows the proposed flow of the additional fund. At the Federal level, the NPHCDA is responsible for transferring funds from the FMOH to the State Primary Health Care Boards (SPHCB), who then disburse funds to Local Government Health Authorities (LGHAs). It is LGHAs that are responsible for funding PHC services in their area.

It has been noted that the LGAs responsible for PHC services have weak capacity and inadequate resources to deliver effective PHC, and the NHAct therefore is an attempt to provide additional funds to PHC. But additional resource allocations through the NHAct will need to be complemented by action to strengthen LGA capacity to deliver PHC services. Formal and informal engagement with key health stakeholders at both national, sub-national and local level by some of the authors have raised concerns over the management of the funds, and the roles of various stakeholders in ensuring accountability for their use. There are still questions about how ready the various stakeholders are to manage this fund since the inappropriate use of the money will lead to difficulty in achieving the objectives of: 1) increased effective funding; and 2) improving the responsiveness of the Nigerian Health System. In order for the flow of revenue from the BHCPF to reach PHC services efficiently, it is necessary that strategies are in place to ensure accountability between the stakeholders at different levels of government as the absence of an accountability lens can actually hamper implementation of the BHCPF and hence health system performance as well as compromise equity.

The overall aim of this study, undertaken before the Bill became law, was to contribute to the generation of policy implementation guidelines around accountability for the BHCPF, by exploring the existing accountability challenges within the Nigerian health system and gathering ideas from health system stakeholders to inform proposals around how to strengthen accountability for the Fund’s implementation.

## Methods

### Study design

This was a qualitative study conducted in Nigeria at the Federal Capital, Abuja and a southeastern state between August 2014 and February 2015. Anambra state was selected for the study because previous work on the effects/impacts of a local accountability structure on health service delivery, resource mobilization and trust by Consortium for Research in Equitable Health Systems (CREHS) had been done in the state and findings from that study was used as an input component in data collection and analysis. These findings included the fact that functionality of a local accountability structure was enhanced by health worker behaviour, and stakeholder supports while de-motivation of health workers, power tussle/social conflict within the community and lack of information about the local accountability structure constrained its functionality [7].

### Data collection and analysis

In-depth interviews with key actors at federal (Policy makers, development partners and donors, civil society organizations) state (Policy makers, directors, programme managers) and LGA programme managers, health Facility heads) levels.

were conducted. Respondents were purposively selected to reflect the different layers of implementation of primary health care and the levels of accountability. To ensure purpose selection, the authors made enquiries from some stakeholders at the different layers of implementation of primary health care on who were likely to be involved in implementing the BHCPF. Then these key stakeholders were selected for the interviews. The authors also relied on their own judgment on the likely key stakeholders based on their knowledge of the participants. Representatives of relevant institutions and offices (National Primary Health care development Agency and National Health Insurance Scheme) who are also key actors that will be involved in implementing the BHCPF were included.

The National Health Bill was first sent to State and LGA officials who were asked to carefully read it. This was followed by interviews with key actors at the national, state and LGA levels to elicit their views on their roles and responsibilities, implementation strategies and accountability process with regards to the Fund. Analysis of initial interview data fed into the next set of interviews, allowing respondents reflect on these findings and react to them.

Information was collected in a cascade starting from the policy makers at the federal level to the actors at the state and LGA levels, in that order. Findings from every level were analysed and presented to respondents at the next level during interviews to elicit their reactions. This was necessary to compare views across levels and allow the testing of different sets of actors’ views with each other. Collecting information ‘in a cascade’ top-down, presents a good approach to exploring perspectives that can help elucidate meaningful information to broaden understanding for policy implementation as policy makers’ viewpoints on issues is “tested” through middle managers and frontline managers. To minimize response bias in this approach of data collection, respondents were first asked questions from the interview guides before findings from preceding interviews were discussed. Interview guides were developed and adapted to different levels and groups of respondents. The questions were structured to elicit respondents’ views about: (i) what they understand their roles in implementation of the Fund to be, and how this compares with their current roles, the challenges/opportunities they envisage in fulfilling these roles and what is clear or not clear in the Bill with respect to their roles; (ii) the mechanisms or strategies they think should be put in place to ensure accountability in the implementation of the Fund, who should be involved, how and to what extent they should be involved, and what linkages should exist between actors; (iii) existing accountability structures, and how these will enable or constrain internal and external accountability in implementing the BHCPF; the challenges in accountability that may be encountered in the flow of funds as stipulated in the Bill; and how best they think the Fund and its accountability should flow.

A total of 24 in-depth interviews and 2 focus group discussions were conducted: 7 in-depth interviews (IDIs) at the national level (including 2 development partners and 2 CSOs); 4 IDIs at the state level; 13 IDIs at the LGA level; and 2 focus group discussions (FGDs) at the ward level. The choice of IDI or FGD for the different respondents was based on appropriateness of the tool and feasibility of its application using previous experiences of interviewing similar actors [8, 9]. Thematic and framework approaches were used to analyse data. Interviews were audio recorded and transcribed verbatim. The transcripts were edited for grammar errors and coded using NVivo software version 10. Information extracted through the coding framework was then analysed.

## Results and Discussions

### Current accountability mechanisms

The existing internal accountability mechanisms at the national level according to the different respondents include a tracking and verification system. This involves the proper documentation of fund that has been allocated for certain activities based on budgetary provision and subsequent retirement of the said funds after expenditures are done. This system, which also has a consumer complaint component, consists of a framework that has been designed to take into consideration all expenditure as well as all income received through, for example, capitation, and re-imbursement. Hence a framework exists to track both income and expenditure, and this was noted by most of the respondents at the federal level. However, one respondent captured it thus in the following quote:

“*we use capitation and fee for service method so … funds can be tracked easily…NHIS does verification to make sure money given to HMO’s reach providers”* (Federal Government Official).

As in the national level, the state government also uses the budget tracking accountability system to monitor expenditure and resource allocation patterns in projects. According to a state government official, this process has been made easier with the establishment of a good governance committee that tracks and monitors budget releases and implementation:

*“there is a good governance committee at the state level, there is a vanguard for good governance committee in every LGA, they also monitor expenditure and budgets to make sure the budgets are being implemented. They track the budgets making sure budgets are being implemented”* (State Government Official).

The state also uses periodic audit checks, public release of funds and community participation as external accountability mechanisms. According to two of the four respondents, the public release of funds entails using different media sources to make budget releases known to the general public; and this enables them to know where to seek for answers when things are not going as expected. The state introduced community participation as an external accountability strategy in which members of the community and religious organizations are involved in budget planning, resource allocation, implementation and monitoring of public health activities in their communities. This fact was noted by all the state respondents, but one respondent noted that:

“*this government is reputed for what we call budgeting forum, every year before the budget comes out, the governor conveys a huge forum, where they ask stakeholders what they want to see in the next year’s budget”* (State Government Official).

Majority of the 13 respondents at the LGA level were in support of the opinion that the state government operates a system of accountability where there are active checks and balances. For instance, a government official stated that the procurement act in the state ensures that no single individual purchases materials for a project; rather, a committee is constituted, with a supervisory arm, to verify any purchases made:

*“we have always checks and balances, and you know in Anambra state, we now use our procurement act. You can’t easily as a single person go to market to make any purchase”* (Local Government Official).

In the local government, the presence of experienced treasurers and auditors was perceived as an important accountability mechanism. These people, they believe, have stayed long enough in the system to know where the loopholes are and how to effectively verify monetary transactions. Equally important is the existence of a decentralized accounting system that consists of local health facility committees and the finance and general memorandum committee for the management of local government funds. This committee works with guidelines that enable them sustain functionality of the LGA accountability mechanism by monitoring and continuous tracking of funds at the LGA level. Other accountability structures mentioned are: (i) supervisory committees such as health committees in the LGA legislature; (ii) funds retirement process; and (iii) use of multiple signatories to LG accounts. Supporting quotes are.

*“We have supervisors, their job is to checkmate. We have the councilors and the committees from the legislatures”* (Local Government Official).

*“It is retiring (of expenditure). Most of the auditors, treasurers ….. are perfect.....they know the best way to handle whatever finance we have”* (Local Government Official).

*“The treasurer is signatory to the account, then the HOD health ….then somebody from the community, so, that there will be proper monitoring and there will be partnership between the community and the LG”* (Local Government Official).

Furthermore, the two development partners at the national and State level stated that they have put in place, systems to ensure judicious use of funds released to the government, and these include: (i) periodic audits of accounts; (ii) fiduciary risk assessment procedures; (iii) diligence assessment which checks governments’ ability to monitor its own contracts and guard against abuse; (iv) spot checks and audits of accounting books and documents. All these have been incorporated into a UN tool called the “National Execution Modalities for Fund management”. These checks are done to establish accountability at the national level and by extrapolation, the sub-national levels. In the words of one of the development partners:

*“what we do with them is diligence assessment but for government what we do is fiduciary risk assessment, before we provide money to government systems”; “we make sure that they not only use them but keep it for audit purposes, so we go in for spot checks, we call it financial program monitoring”* (Development Partner).

#### Current challenges in accountability

A majority of the respondents stressed that there were issues with trust, transparency and corruption in government especially where it concerns fund management. Almost all respondents opined that corruption within the health system is one major barrier to success of the BHCPF. As noted by a respondent “*like you know the issue of corruption also exists in the health system among health workers including those at the health facility especially those who sell government drugs and LGA level who collect money before posting you to a health facility……..In fact it is everywhere in the health system* (Local Government Official).

Corruption in the health sector has made various health institutions to be ineffective while scarce resources invested in the sector are wasted. Health system corruption prevails in Nigeria among different actors including senior and junior administrative officers in health ministries, parastatals and agencies, health officials and among political office holders. This is because there is no adherence to the rule of law, coupled with lack of transparency and trust. In addition, the public sector in Nigeria is ruled by ineffective civil service codes and weak accountability mechanisms, among others [10].

Interference at higher levels of government also contribute to making funds inaccessible to implementers when they need them, and the result is that funds are not utilized for planned activities. According to most of the State respondents, the federal level officers are usually more interested in organizing workshops where they are expected to be paid per diems and DSA or more interested in procurement activities with attendant kickbacks. As a result, they are more inclined to releasing money to such activities at the expense of releasing money to ensure access to health services. This was captured thus:

*“Some officers from the Ministry of health and even at the federal are just interested in going from one workshop to another collecting per diems and that’s all…” (State Government Official).*

*Some Federal people are more interested in the commission they will collect if they issue out contracts for jobs…..(State Government Official).*

*“The FMOH, should show the stewardship role ….to ensure that we do not create another source for procurement or doing only conferences with the money. So that the money will be targeted to … providing access to health care”* (State Government Official).

Overall, political interests interfere with primary health care resource allocation and distribution in Nigeria and in terms of health distribution, historically and up till now, they are highly dependent on political influences and peoples’ influences [11]. There is the tendency for political office holders to attract development projects, including PHC infrastructure and services, to their own locality regardless of need*.* Political interests also influence programme implementation for example according to a respondent, “*if you ask for local workers to participate in national or state immunization programmes they will only submit their relations whether they are qualified or not. They even influence who is posted to the health centers”* (Local Government Official).

Also, some politicians have been seen to pay attention to health services or programmes that are either high on the global or national agenda, or for which funding is available, or that would guarantee immediate results and electorates’ support.

Poor data management constitutes a challenge to accountability because data are needed to make decisions and plan. Majority of the respondents feel there is hardly any data collection taking place, and where there is it is neither easily available nor reliable Although the current information management system in Nigeria is deemed adequate in terms of structure of collation and transmission, there are notable shortfalls in its ability to deliver timely, reliable and complete data for several reasons. For example, a respondent noted that “*there are so many different types of forms in the facilities which the health workers are expected to fill… you have for malaria, you have for TB, you have for HIV, you have for pregnant women…….they do not have that kind of time to fill them”* (Local Government Official). Furthermore, the culture of routine analysis of the data and feedback to health institutions, and its use for health planning and improvement of health outcomes is yet to take root [12].

The capacity to collect, collate and analyse data is perceived to be relatively poor at the primary care facility level compared to higher levels of reporting and service delivery. However, it was stated that health information management for vertical disease control programmes such as HIV and malaria is more effective at generating reliable data than the integrated system. Consequently, health planning and priority setting are not based on evidence of the accurate epidemiological profile of the population.

The system is equally completely without information on services delivered in the non-state sector and lacks the mechanism to capture such data. This was captured by a respondent thus: “*self-treatment or treatment from traditional and religious healers typically go undocumented by health workers”.* (Local Government Official).

The weakness in the system is reflected at all levels of the health system and has been attributed to weak governance and poor commitment to duty, poor funding and infrastructure, weak capacity and inadequate personnel and skills in data management. In addition, sharing financial information in Nigeria is a very sensitive issue and there is a lack of political will to share financial data and lack of financial information is widespread, especially at the LGA level.

Political commitment from the State in relation to release of funds for health activities was also mentioned as a challenge by most of the respondents, especially where implementation requires multiple political and bureaucratic stakeholders. The long waiting period from when the budget is announced and when actual release of funds is made was said to be a major problem because it undermined implementers’ ability to plan appropriately for their activities.

One respondent observed that where it concerns the BHCPF, “*technocrats have to depend on politicians to provide the counterpart fund, and if the politicians do not support the bill, technocrats will not be able to do much in implementation”* (State Government Official)*.*

Poor motivation, mentorship, monitoring and supervision were cited as key challenges to health workers’ performance and that in order to foster a sense of responsibility and accountability in the staff there needs to be: (i) a reward system for performance; (ii) institutionalized mentorship to give workers a sense of fulfillment; and (iii) strengthened supervision and monitoring of activities to be able to detect staff who are derailing to put them back on track:

*“There has to be check and balance. You don’t just give somebody money and leave him to do whatever he likes. You should be inspecting what he is doing to know whether he is derailing, whether he is doing the actual thing he is asked to do”* (Federal Government Official).

Respondents at the state and LGA levels expressed concern about the failure of past financial reforms to achieve set goals as a result of mismanagement and diversion of funds. At the LGA level, it was stated that some government officials might see the BHCPF as an opportunity for them to accumulate wealth for themselves, and they could deliberately work against the accountability system by ensuring that the mechanisms put in place to ensure accountability are not implemented. A respondent stated that a bottleneck to proper accountability could arise from the states if the money meant for the local government is not provided completely or at the right time. This of course will impact on the planning and efficient utilization of funds to provide needed services at the local government level. There was a consensus opinion that the absence of guidelines for managing the funds could present a challenge to implementers, among who are poorly trained and incompetent managers:

*“So, there should be a system (guideline) on ground to carry out these programmes. I told you that the primary health care has a big challenge as far as manpower is concerned because in some places, we have incompetent people managing the system; who are not very knowledgeable about what they are doing”* (Local Government Official).

The key challenges outlined by development partners at both federal and state level are: (i) weak financial management and accountability systems in government which deter donors from funding the Nigerian healthcare system; (ii) weak capacity to implement suggested accountability mechanisms due to political interference with accountability structures; (iii) tendency of government officials and politicians to pursue their personal interests. Some supporting quotes are:

*“Because that fund is going to be contributed by many, there will be many players. … the fiduciary arrangement around it needs to be very robust because currently many donors are not able to put funding into government systems in Nigeria. For that vision in the Bill to actually come to reality, the accountability system for that Fund will have to be much stronger than there is in Nigerian environment”* (Civil Society Organization).

*“Government systems are designed to hold government accountable, it is just implementing it, what we should just be looking for is how can we force their hands to adhere to the system”* (Civil Society Organization).

### Proposed accountability mechanisms for the BHCPF (external and internal)

Five (5) accountability levels and key stakeholders expected to implement the BHCPF are:

1. The Federal government (Federal Ministry of Health, NPHCDA, NHIS, Federal Ministry of Finance-FMOF);
2. The State government (State Ministry of Health, SPHCB, State Ministry of Finance, Ministry of Local Government);
3. The Local government (LGHA);
4. PHC frontline Health facilities (Health workers, Health facility committees and
5. External actors (Development partners and donors, CSOs, Community members).

**Fig. 2:**
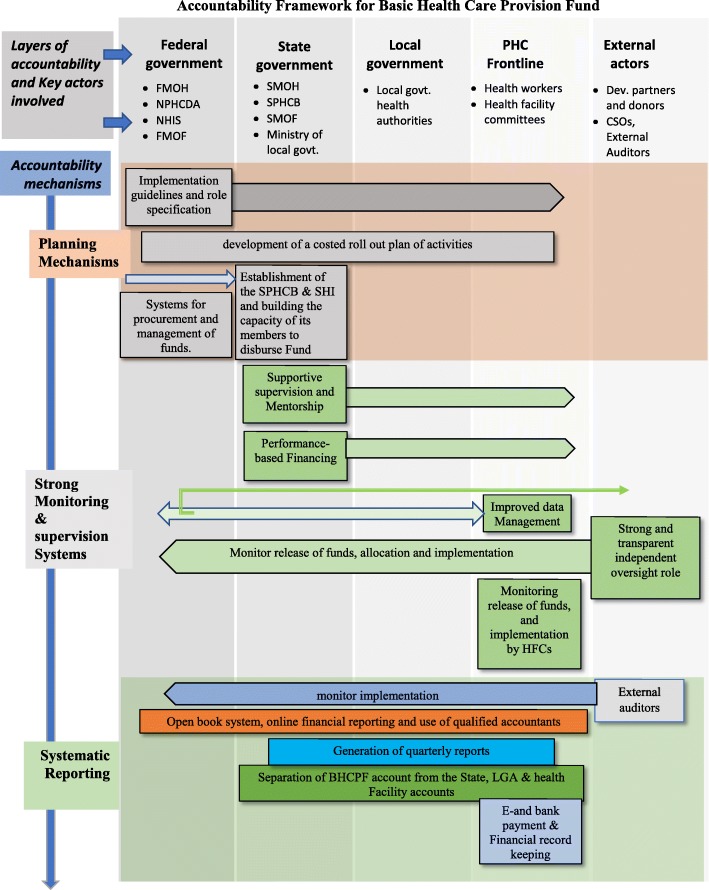
Accountability Framework for BHCPF

To ensure accountability of the proposed BHCP fund, an overall accountability framework for its implementation has been proposed (Fig. 2). In developing this framework, the authors took into consideration the views of the different actors regarding the current challenges in accountability, as well as their ideas on how to address them in future. The framework is proposed as a coherent set of mechanisms that must be implemented at and across the different levels of the health system, working together to ensure accountability. They encompass planning mechanisms, strong and transparent monitoring and supervision systems, and systematic reporting at different levels of the healthcare system.

It is hoped that this accountability framework will add to the accountability literature and help in dealing with some of the current accountability challenges including trust and transparency, interference at higher levels corruption in the health system, poor data management, poor motivation, mentorship, monitoring and supervision of health workers.

#### Planning mechanisms

This planning mechanism involves development of implementation guidelines, development of a costed roll out plan of activities, establishment of the State PHC Boards (SPHCB) and State Health Insurance Scheme (NHIS), building the capacity of State and Local Government Health Authorities to disburse Fund and systems for procurement and management of funds.

##### Development of implementation guidelines

Several national level respondents were of the opinion that the federal ministry of health should develop clear guidelines about who is responsible for implementing the fund and what their roles will be. When these are developed, they should be used by the State, LGA and PHC frontline health workers as shown in the arrow in Fig. 2. They suggested that specification of tasks and responsibilities of implementers at the state and local government and frontline PHC levels would enable accountability since people will better understand their roles and what is expected of them at every point in time. The use of implementation guidelines was stated as a means of achieving this, thus streamlining the activities of the state and LGA staff. According to one of them:

*“There is this responsibility. Everybody is alive to it, and guidelines must be circulated so that everybody at any point knows what they are doing”* (Federal Government Official).

“*things don’t usually happen the way you want them to unless you outline them…….and when specific guidelines and regulations are documented, it’s easier to measure outcome”* (Civil Society Organization) *.*

One of the reasons for the Nigerian weak health system is the lack of clarity of roles and responsibilities among key stakeholders at the different levels of government [13]. And as noted elsewhere the clarity in the roles of stakeholders and implementers, and the nature of relationships between key actors, are recognized as critical to policy implementation [14, 15].

Some authors have stressed that ambiguity of roles was related to confusion among stakeholders about the objectives, scope of practice, responsibilities and anticipated outcomes of the roles of stakeholders [16]. Variable stakeholder awareness and competing stakeholder expectations also has been noted to contribute to a lack of role clarity [17]. According to the authors, when the role means different things to different people and there is lack of consensus about role expectations, role conflict and role overload can occur. Therefore, it is expected that clarity of roles and responsibilities through production of appropriate implementation guidelines will enhance both internal and external accountability measures.

Furthermore, concerning the implementation guideline, there was a consensus across all levels that since it will be a national document, it must be communicated in such a way that all implementing states and LGAs would understand and find useful to guide their plans and actions. This was captured by a respondent thus:

*“It is very important that the guideline is well spelt out….such that the person in Lagos will understand it as clearly as the person in Adamawa or Borno State. It will be a national guideline that everyone can fit”* (Federal Government Officer).

Communicating the guidelines is very important as studies have shown that inability to communicate policy and guidelines to health program implementers resulted in misinterpretation of health programs [18]. Due to limited policy dissemination and awareness, many stakeholders are usually uninformed regarding the policy objectives and implementation strategy and the agencies responsible for implementation. The limited health communication in the context of changing healthcare environments and diverse populations is also an important underpinning of rising healthcare costs and sustained health disparities. And as has been noted elsewhere, the key issue for communicating strategy should be able to align the extent and scope of the change in healthcare environment and the approaches of implementation of the changes with the values and principles outlined in the related policy document in question [19].

Most respondents felt that all levels of government, but particularly the implementers, need to sustain their commitments towards achieving the Fund’s goals, and that this would be made easier for them if they are clear about their roles and the limits to them.

##### Development of a costed roll out plan of activities

The development of a costed roll out plan of activities by the federal government, State, LGA and PHC frontline health workers was proposed by a majority of the respondents as a good approach to managing the funds. This would first involve the development of a plan of all the activities needed within a certain period, followed by periodic budgets based on the plan and then a justification of how the money was spent with accompanying evidence and outputs to show. This is expected to serve as the basis for resource allocation and mutual accountability by all stakeholders – government, development partners, civil society and communities. Reference was made of the success of a previously funded project that had been carried out by the local government using this approach:

*“we can do what we call redraft the plan, you make a roll out plan for the activities and that roll out plan we cost it, after costing it, you see the specific area the money will go”*.(State Government Official).

To operationalize the costed plan, it was suggested by respondents that at the federal level a committee should be constituted to ensure that BHCP fund is captured in the budget.

*“an Inter-Ministerial Committee on Innovative Financing should be formed to ensure that the 1% CRF is appropriated in the budget”* (Civil Society Organization). This is particularly important because despite the signing of the bill in 2014, the 1% CRF was not captured in the 2016 and 2017 budget. According to the respondent, the formation of this committee will ensure this does not happen in future. This committee will also ensure that guidelines, manuals & strategic plans are developed at the national level.

At the State and LGA levels, the costed plan of activities was to be submitted for access to the stipulated fund which will enable proper accountability. And to operationalize the plan, at the State and LGA levels, the stakeholders outlined in the proposed accountability framework at these levels (SMOH, SPHCB, SMOF, Ministry of Local Government) should ensure that the counterpart funding of 25% is captured in the budget, annual State and LGA operational plans and is drawn and reflected in the medium-term expenditure framework as well as medium term sector strategy.

##### Establishment of the state PHC boards (SPHCB) and state health insurance scheme (SHIS) and building the capacity of members to disburse fund

The States should establish the SPHCB which is a requisite for benefiting from the fund and the NHIS. These institutions should be backed by laws enacted by the State Legislators. The SPHCB should then develop their strategic health development plan which should embrace the concept of one management, one plan and one monitoring and evaluation for PHC in the State otherwise referred to as “PHC Under One Roof” (PHCUOR). This concept is modelled on guidelines developed by the World Health Organization for integrated district-based service delivery to strengthen PHC services through reducing the fragmentation of PHC service management [20]. However, an issue in the fund’s institutional arrangement, which state and LGA level respondents were concerned would be a major challenge to implementation is who heads this SPHCB. The reason this could be an issue is that the direct implementers of PHC activities, and consequently the Fund, are the LGAs who will also be contributing a significant amount to the states counterpart. Meanwhile, the Bill appears to be silent on LGA level arrangements for managing the Fund. As captured by a respondent:

“*The truth is there have been hiccups here and there about setting up that board. It is like who is going to be in control, ….the LG expect that they being the providers of most of the fund (LGA counterpart) they should be in control”* (Local Government Official).

It was also suggested that a legal framework where funders and implementers sign an agreement gives the state a sense of responsibility and commitment. As captured by a respondent:

*“Having a legal frame work … which they are going to sign; some of them will now resign if they don’t have good intentions“*(Local Government Official).

Building the capacity of the members of the SPHCB and SHIS to disburse Fund revenue effectively was stressed as part of the planning for accountability. It has been observed that, “regardless of the precise nature of a policy or strategy, and the support that exists for it, if the means to implement it are inadequate in terms of capacity or capability, or both, then it will count for little” [21]. However, one of the major criticisms of capacity building is that it is a ‘top-down’ approach that is often linked to a government’s agendas for change. But, Fitzgerald makes the point that this can also be a strength [22]. He argues that when initiatives are supported and reinforced by ‘systems’ they are probably more likely to be sustainable. Therefore, building the capacity of the States and LGA by the federal should be seen as a strength in the fund management and this can be reinforced from time to time to ensure sustainability.

##### Systems for procurement and management of funds

All the development partners interviewed were of the opinion that the federal government should establish a robust system of procurement and management of funds. It was argued that this will enhance internal accountability and minimize corruption. The use of strategic procurement systems in the purchase of drugs and other essential commodities was suggested as a means of achieving better value for money having made an observation that the cost of pharmaceuticals in Nigeria is more than it ought to be. This was captured by one of the development partners thus:

*“I think that there is a big opportunity to look at the procurement system … and be more strategic about procurement. Because if you look at the cost of pharmaceuticals in Nigeria, it is way beyond what it should be, and actually we could get a lot better value for money when we use a strategic procurement system”* (Development Partner).

#### Strong monitoring and supervision systems

This involves the institutionalization of a supportive supervision and mentorship programs, performance-based financing, Strong and transparent independent oversight role and improved data management.

##### Supportive supervision and mentorship

In general, supportive supervision is a process of guiding, monitoring, and coaching workers to promote compliance with standards of practice and assure the delivery of quality care service. The need for constant and supportive supervision and monitoring by the State as well as mentoring and training on how to address specific challenges was emphasized by majority of respondents at the LGA level as a good accountability strategy.

The Nigerian national strategic health development plan recognises the need to establish and institutionalize a framework for an integrated supportive supervision with adequate committed resources for all types and levels of care providers across public and private sectors [23]. This could be leveraged by the fund managers. Some of the State and LGA respondents were also of the opinion that supportive monitoring and supervision for health workers at the facility by the LGA would guard against waste and prevent health workers in the facilities from managing the BHCPF in the ways they deem fit. This is very important because from LGA responses, systems are actually in place to ensure accountability within the context of systems for procurement and management of funds. However, compliance is the main issue, and this was echoed by development partners. So supportive supervision and mentorship is likely to address and promote compliance. Thus, as shown in the framework, the State will supervise the LGA and the LGA will supervise the PHC frontline. The institutionalized mentorship is likely to give workers a sense of fulfillment while strengthened supervision and monitoring of activities will be able to detect staff who are derailing and put them back on track.

It was recommended that the use of reward and mentorship-based monitoring and evaluation system comprising of different levels of participants, including community members, would increase community participation, ensure fund management compliance and improve accountability for the Fund. This system would be such that states that are not doing so well are mentored and trained on how to go about addressing their specific challenges. The need to discuss the evidence documentation and reports pertaining to implementing and managing the funds at all levels, and with the community, to encourage participation and foster transparency in the management of the fund was also noted*:*

*“You can just do a report and that will end in my office. But there should be a process of socializing, breaking the evidence at all levels: local, community and all”* (State Government Official).

##### Performance-based financing

Most of the national level and development partners’ respondents, as well as some local government officials, believed states and LGAs should institutionalize the practice of results-based financing both as a reward system and as an accountability system. Here, new disbursements are conditional on the results of previous disbursements and these results could be monitored using some performance indicators. This should be developed at the State level and implemented at the LGA and PHC frontline as depicted in Fig. 2. They believed that to address issues of accountability at any level especially the local government level, performance indicators should be tied to disbursement of funds. They also suggested that peer-review mechanisms, and healthy inter-state and inter-LGA competitions could motivate states and LGAs to want to increase their output to get more funding. These were captured by some of the respondents thus:

*“I think that for the PHC fund, there needs to be performance indicators that needs to be tied to the disbursement of this fund, because if not, if we don’t address the issues of accountability, it is like putting money into the black hole”* (Local Government Official).

*“The concept of results-based financing is coming to play here, and if the states get used to it, there will be more accountability and transparency”* (Federal Government Officer).

*“I think that for the PHC fund, there needs to be performance indicators that needs to be tied to the disbursement of this fund, because if not, if we don’t address the issues of accountability, it is like putting money into the black hole”* (Development Partner).

Results Based Financing (RBF) – also known as Performance Based Financing (PBF) or Payment for performance (P4P) – involves the payment of financial rewards to health facilities or health workers based on their achievement of performance targets. Many experts see the introduction of RBF schemes as an opportunity to strengthen, or even reform, health systems; however, they are also a potential source of new risks and challenges that are not well understood [24].

RBF can enhance accountability and improve health information systems since reported results are verified when linked to incentives. Local authorities verify results, which are publicized for each organization or health facility. A rigorous impact evaluation of the RBF scheme in Rwanda demonstrates that RBF can have strong positive impacts [25], but our understanding of why RBF has such positive effects in Rwanda is still very limited, and thus it is unclear whether such schemes could have a similar potential in other settings. Other authors have also recorded some successes with RBF in Tanzania [26]. It has also improved both external and internal accountability in a Tanzanian pilot study [27]. Although the RBF does not directly attack the overall environment of corruption, it is a way of promoting efficient public service delivery, offering less incentive or opportunity for diversion of funds than current payment systems [28]. However, some authors have noted the potential risks of RBF in relation to non-targeted service use [29].

In Nigeria PBF is being implemented in 3 states of Nigeria- Adamawa, Nasarawa and Ondo by by NPHCDA and the SPHCDB of the 3 states under the Nigeria State Health Investment Project (NSHIP). It aims to improve health results by providing health facilities autonomy and make them accountable and motivated for results. Under NSHIP, roles of the states and LGAs are clearly defined with their result indicators, and the financial incentives are provided on the achievement of the indicators. PBF also provides direct finance to health centers based on the quantity and quality of services delivered, and the PHCs have autonomy in using it to improve health services [30].

Although other types of inputs do often accompany PBF reform packages, like capacity building through training, mentoring and supervision, and coaching and technical assistance on measurement, use of improved management tools and how to increase results, a key challenge for instituting PBF for the BHCPF will be data management as the current information system is poor with lack of capacity at PHC frontline health facilities to implement it. So, for PBF to be effective for the BHCPF, there is need to improve and strengthen the information system and build capacity of PHC frontline health workers.

From a funding point of view, sustainability is likely to depend on a combination of government buy-in and continued external support as all PBF packages in Nigeria are highly dependent on external support.

##### Strong and transparent independent oversight role

The Oversight of the BHCPF recognizes the huge investment by the Government of Nigeria (GoN) towards the improvement of PHC. It further recognizes the need to ensure that there is ‘value for money’ and most important that Nigerian communities, the primary beneficiaries, derive the expected benefits from the Fund. Government agencies require objective oversight of any major technical initiative to ensure that projects are being properly managed, resources are being properly deployed and goals are being met.

In this regard, effective systems for oversight of the implementation of the Fund is proposed to be put in place to ensure periodic accountability and progress reporting to the federal government. The objective is to provide independent oversight and validation at all levels; federal, state, local government and PHC frontline, of the Fund’s implementation, monitor progress and ensure delivery on the targets. This oversight function should be carried out by development partners and CSOs as shown in Fig. 2. Having an independent and unbiased partner in the BHCPF’s success will give leadership valuable insight into potential risks, areas for improvement and even “blind spots” in the internal governance and management of the Fund. It will also ensure accountability.

The involvement of Community members, through Health Facility Committees, in decisions regarding how health facility revenue is spent is also important. Active community participation was emphasized by majority of respondents at the LGA level as the presence of community members for example will help contain government excesses.

*“Community members should be involved because they will serve as the watch dog”* (Local Government Official).

Thus, involvement of community members in the release of funds for primary health care activities was perceived to be a useful strategy for ensuring accountability since they can monitor how the funds are being spent. These were captured by the following quotes:

*“If any money is being released, for primary healthcare projects, the Vanguard committee on good governance within the communities should be notified about funds”* (State Government Official).

However, a dissenting voice was of the opinion that involving community members would be a mistake because multiple interests are represented, and this is difficult to manage. According to him,

“*the lowest individual in the community is always looking for money and if they know money is coming they will like to get their own share thinking of their own interest and so the goal will not be achieved”* (Local Government Official).

The community members through the health facility committees usually helps to raise awareness on health disparities issues, and they create a link between communities that are often underserved and facility as well as the legislative members (councillors) that represent the communities’ health needs. This is important in helping move towards health and social equity as well as greater accountability.

##### Improved data management

Part of the challenges of current accountability system as reported by the respondents is poor data management. Most of the challenges in data management according to the Nigerian health systems assessment report 2014, are in the areas of data governance, data quality and use of Information. Therefor the proposed accountability mechanism involves the institution of good data management system. Effective and efficient planning, monitoring and evaluation of health services depends on reliable data. This will help to monitor the progress towards stated goals and targets of the BHCPF. The information process will involve data collection and analysis at the community and PHC level and transmission of such information to the LGA, State, federal and development partners as shown in the framework. There is also a feedback of the information to the health facility.

Specific intervention for data management should involve development of state M&E framework, ocnduct M&E/HMIS training for LGA and facility staff, provide adequate tools to health facilities and conduct regular data quality assurance. Thus, State governments should print and distribute to LGAs and health facilities data tools and data equipment. Capacity building of health workers on the tools should be of priority for effective data collection. Routine data quality assessment should be conducted to validate data generated.

#### Systematic and transparent reporting

This entails external auditors to monitor implementation, separation of BHCPF account from the State LGA and facility health accounts, use of electronic and Bank payment, external auditors, open book system and online reporting and generation of quarterly reports.

##### External auditors to monitor implementation

At the national level, the level-specific accountability strengthening strategies would be the use of external auditors to monitor and evaluate their financial activities across both federal, State, LGA and frontline levels, and publication of financial reports about the BHCPF on their websites for ease of access. It was also suggested to consider making dispersal of revenue from NPHCDA to SPHCB conditional on the results of previous disbursements.

The CSO respondents were of the view that in order to improve the accountability system in government at all levels, there should be an open-book policy, where the government’s accounts and books are open to auditing by the public and external auditing by CSOs, non-governmental organizations (NGOs), and community groups is encouraged:

*“I think external auditors in the form of community, CSOs, NGOs should be part of the monitoring and there should be openness and auditing and publication”* (Civil Society Organization).

##### Separation of BHCPF account from the state LGA and facility health account

Separation of BHCPF from State, LGA health account was suggested on the premise that it will make record keeping easier and management better and therefore ease of accountability. Also, at the facility level, it was suggested that a separate BHCPF account should be opened for the same reasons and community members involved in its management.

The success of having a separate facility account and involving the community in fund management has been documented. For example, in the HSSF in Kenya central funds are credited directly into a facility’s bank account quarterly, and facility funds are managed by health facility management committees including community representatives. An evaluation of the programme showed that HSSF funds were reaching facilities; funds were being overseen and used in a way that strengthened transparency and community involvement. It also improved health workers’ motivation and patient satisfaction [31].

However, there were challenges – such as complex and centralized accounting requirements wider negative impacts (in Kenya, difficulties for facilities in accessing crucial user fee funds), and lack of clarity in the roles and responsibilities of key actors [31]. Perhaps most critically, given the potential for misappropriation and misuse in peripheral facilities [32], there is a need to balance fiduciary oversight with administrative and monitoring burdens as has been noted by some authors [33].

##### Use of electronic and Bank payment, external auditors, open book system and online reporting

Respondents at the state and LGA level felt that the current auditing systems are too porous and highly bureaucratic and would benefit from reinforcements. There was a suggestion that using electronic and bank payments for transactions between consumers and service providers would make the system more transparent by eliminating the middlemen who jeopardize the whole system, and promote good record keeping. It would also improve trust and accountability in managing the Fund and hence reduce corruption. And as noted by some of the respondents:

“*The community banking process at PHC level should be initiated so as to keep a record of fund management at this level”* (State Government Official).

*“Any payment in this fund should be e-payment. That is the only thing I want to suggest… e-payment reduces fraud”* (Local Government Official).

It was also proposed that credible honest individuals who have the appropriate qualification should be employed at federal, state, LGA and frontline levels. Thus, the use of qualified and experienced accountants and use of external auditors and publishing financial reports on SPHCB websites in accordance with the Freedom of Information and Fiscal Appropriation Act were suggested. They opined that if financial reports of government are made publicly accessible through the internet, people can access the information using their mobile phones. This would encourage transparency and reinforce accountability and ensure equity. A respondent captured it thus:

*“there needs to build an accountability relationship with the general public…..you can have a financial report put online, such that anybody can go and access that information and everybody will be in the know on what is happening”* (Local Government Official).

*“there is too much corruption in Nigeria now. Money disappears at every level … but you can check this by employing people who are basically honest, and if you make them comfortable, they can keep their hands off public money, so it really matters”* (State Government Official).

However, the manner by which qualification would enable accountability was not stated by respondents, but the rationale could be that people who are trained for a particular position and have attained a certain level of education would better understand the scope of their jobs and appreciate the need for an effective health care system.

The CSOs respondents on the other hand were of the view that to improve the accountability system in government at all levels, there should be an open-book policy, where the government’s accounts and books are open to auditing by the public and external auditing by CSOs, NGOs, and community groups is encouraged. They were also of the opinion that specific guidelines for implementation of this would ensure that things are done right.

*“I think external auditors in the form of community, CSOs, NGOs should be part of the monitoring and there should be openness and auditing and publication”* (Civil Society Organization).

##### Generation of quarterly reports

At both State, LGA and facility level, quarterly reports should be generated based on all the activities including financial and activity reports. The report should include successes and identifying obstacles to progress and limitations for all the proposed timeliness as in the costed plan with corrective actions attributable to named individuals. However, the capacities of these actors need to be built on delivering these critical actions because building and sustaining a narrative for health equity requires capacities to make these documents and decision processes/outcomes available.

A government official perceived that a good reporting system would be essential for better accountability, and suggested that reporting tools and aids should be made easily accessible to implementers:

*“the tools for reporting should be placed at points where they are required so that if you want to report it shouldn’t be that you don’t have the tools”*.(Federal Government Official).

The report should include utilization updates on drugs, vaccines, immunization etc. In doing this it is important to note that the time taken for health workers to fill these tools could eat into the time used by them to see their patients and efforts should be made to reduce this and unmanageable paperwork is avoided. In the health sector services funds (HSSF) study in Kenya many interviewees reported that completion of required reports took significant amounts of in-charges’ time [31].

## Conclusion

In general the respondents were of the opinion that the goals of the fund, could be achieved on the conditions that there is: (i) a written, well communicated implementation guideline for the fund; (ii) sustained political commitment; (iii) transparency of the implementing actors; (iv) establishment of the SPHCB with clear responsibilities for the state and local government; (v) proper awareness and education of both users and providers; and (vi) timely release of fund. Most respondents felt that all levels of government, but particularly the implementers, need to sustain their commitments towards achieving the Fund’s goals, and that this would be made easier for them if they are clear about their roles and the limits to them.

Critical, current challenges of health care accountability in Nigeria include trust, transparency and corruption in the health system, political interference at higher levels of government, poor data management, lack of political commitment from the State in relation to release of funds for health activities, poor motivation, mentorship, monitoring and supervision, weak financial management and accountability systems and weak capacity to implement suggested accountability mechanisms due to political interference with accountability structures.

These challenges highlight the need for careful and comprehensive action to safeguard the value of the new Basic HealthCare Provision Fund and ensure that newly available resources are used for the intended purpose. This paper has proposed a framework to guide the development of the implementation of strengthened accountability for the BHCPF, based on understanding of current challenges and multiple actors’ views of how to address them.

Critically, the framework offers a system-wide approach to strengthening accountability - working across all tiers of the health system, engaging multiple actors and involving multiple mechanisms. It is not enough to introduce a mechanism at LGA or state level, and it is absolutely essential to work at every level and cascade mechanisms up and down levels and work with external actors and internal actors. Strengthening accountability demands action across the system as a whole, working with financing, performance and political accountability and combining external and internal mechanisms.

Furthermore, it is important to monitor the usage of the BHCP Fund at the federal, state, and local levels which should be conducted regularly to ensure equity in healthcare provision. This is essential because health equity is not very high on the policy agenda, thus creating a problem of affordability and accessibility among the poor and the less advantaged groups in Nigeria.

### Limitations of the study

Our sample of stakeholders at all levels was not intended to be representative of the whole country, but rather those who have a practical range of views from their experience with the Nigerian health system. The views of the media in accountability structure and mechanisms were not evaluated in this study. This will form a basis for a further study.

The results of this study cannot be generalized to other settings because of the various contextual factors that may play out in those settings including their level of health systems performance and availability of interventions to strengthen the health systems.

Finally, this paper did not set out to present a fully conceptualized framework, but instead, initial ideas towards such a framework. However, the value of the paper lies in presenting the findings of the inquiry that led to this framework and this framework has been used by policy makers as a valuable guide for the development of operational plan for the implementation of the Basic HealthCare Provision Fund (BHCPF) by the Federal Ministry of Health and other key stakeholders [34].

## Abbreviations

BHCPF: Basic Healthcare Provision Fund
CREHS: Consortium for Research in Equitable Health Systems
CRF: Consolidated Revenue Fund
CSO: Civil society Organization CSO
DP: Development partners
FGD: Focus Group Discussions
FMOF: Federal Ministry of Finance
FMOH: Federal Ministry of Health
HFC: Health facility committees
IDI: In-depth interviews
LGA: Local Government Authority
LGHA: Local Government Health Authorities
MTEF&FSP: Medium Term Expenditure Framework and Fiscal Strategy Paper
NDHS: National Demographic Health Survey
NGO: Non-governmental organizations
NHAct: National Health Act
NHIS: National Health Insurance Scheme
NPHCDA: National Primary Health Care Development Agency
NSHIP: Nigeria State Health Investment Project
P4P: Payment for performance
PBF: Performance Based Financing
PHC: Primary Health Care
PHCUOR: PHC Under One Roof
RBF: Results Based Financing
SMOF: State Ministry of Finance
SMOH: State Ministry of Health
SPHCB: State Primary Health Care Boards
U5MR: Under 5 mortality rate
